# YOLO-CBD: Classroom Behavior Detection Method Based on Behavior Feature Extraction and Aggregation

**DOI:** 10.3390/s25103073

**Published:** 2025-05-13

**Authors:** Shuyun Peng, Xiaopei Zhang, Luoyu Zhou, Peng Wang

**Affiliations:** 1 School of Electronic Information and Electrical Engineering, Yangtze University, Jingzhou 434023, China; 2023710651@yangtzeu.edu.cn (S.P.); wangpeng286473@163.com (P.W.); 2 Electrical and Computer Engineering, University of California, Los Angeles, CA 90095, USA; zxpmirror1994@g.ucla.edu; 3 Institute for Artificial Intelligence, Yangtze University, Jingzhou 434023, China

**Keywords:** classroom behavior detection, feature extraction, feature aggregation, multi-scale feature fusion, YOLOv10

## Abstract

Classroom behavior can effectively reflect learning states, and thus classroom behavior detection is crucial for improving teaching methods and enhancing teaching quality. To address issues such as severe occlusions and large scale variations in student behavior detection, this paper proposes a classroom behavior detection model, named YOLO-CBD (YOLOv10s Classroom Behavior Detection). Firstly, BiFormer attention is introduced to redesign the Efficientv2 network, leading to a novel backbone network for efficient feature extraction of student classroom behaviors. The proposed attention module enables accurate localization in densely populated student settings. Secondly, a novel feature aggregation module is designed for replacing the basic C2f module in the YOLOv10s neck network and enhancing the capability to detect occluded targets effectively. Additionally, a feature pyramid network with efficient feature fusion is constructed to address inconsistencies among features of different scales. Finally, the Wise-IoU loss function is incorporated to handle sample imbalance issues. Experimental results show that, compared to the baseline model, YOLO-CBD improves precision by 5.7%, recall by 3.7%, and mAP50 by 3.5%, achieving effective classroom behavior detection.

## 1. Introduction

The rapid development of artificial intelligence is driving various industries toward intelligent transformation [1]. Smart classrooms, as a new teaching model, are increasingly applied in the education field. Assessing and analyzing classroom behavior is a vital component of smart classrooms [2]. Classroom behavior not only reflects students’ learning states, but also indirectly verifies the teaching quality. Observing and evaluating students’ classroom behavior helps identify deficiencies in the teaching process and allows for targeted interventions [3]. Therefore, this study focuses on detecting and analyzing students’ behavior throughout the classroom, aiming to provide a reference for research on classroom behavior under smart education.

Student classroom behavior detection is essentially a target detection task in computer vision [4]. Traditional classroom behavior detection methods rely on manually observing surveillance videos, which are often limited by inefficiency, low accuracy, and observer fatigue [5]. Consequently, researchers have explored classroom behavior detection technologies based on deep learning [6,7,8] and other methods [9,10,11].

In recent years, with the rapid development of deep learning technology, deep learning-based algorithms for classroom behavior detection have gained widespread application. These algorithms can be generally divided into two-stage and one-stage categories. Two-stage algorithms first generate a series of candidate regions, followed by precise classification and localization adjustments. Representative algorithms include R-CNN, Fast R-CNN, and Faster R-CNN [12]. In contrast, one-stage algorithms directly predict the category and location of the target on the feature map, achieving both high detection accuracy and significantly enhanced detection speed. Representative algorithms include YOLO series methods [13,14,15,16,17,18,19,20] and SSD [21]. However, in complex classroom environments with dense occlusions and multi-scale target variations, the existing algorithms tend to produce false positives and miss detections, thereby increasing the difficulty of classroom behavior detection.

To reduce the influence of irrelevant backgrounds, Li et al. [22] proposed an improved SlowFast model for student behavior recognition. However, the model has a high parameter quantity and slow detection speed. Wang et al. [23] proposed a novel SLBDetection-Net behavior detection method, which introduces a specific attention mechanism to prioritize the perception of learning behaviors. However, the algorithm’s complex structure and long detection time limit its suitability for real-time classroom behavior analysis. To address student occlusion issues in classroom monitoring, Zhao et al. [24] proposed an improved Transformer-based student behavior detection model, which uses Bi-FPN to effectively fuse features from different scales. However, this model is sensitive to data variations and noise, reducing recognition accuracy. Liu et al. [25] presented PV-YOLO, a model that adopts RFAConv as the backbone, BiFPN in the neck, and a lightweight detection head, enhancing detection accuracy for small targets. Jiao et al. [26] proposed RS-YOLO, an algorithm that used MS-PAFPN, WS-Fusion, and SPPFormer to improve semantic feature representation and interaction, achieving excellent performance in benchmarks. Zhao et al. [27] introduced CBPH-Net, a one-stage classroom behavior recognition method that enhances multi-scale recognition capabilities by using convolution kernels of different sizes and multi-scale features, reducing small-target information loss during sampling. However, the multi-scale feature nodes increasement raises computational costs.

In summary, in the task of dense classroom behavior detection, large target scale differences and occlusion are the main factors that limit the accuracy of student classroom behavior detection. To address these issues, this paper proposes a classroom behavior detection model. The main contributions of this paper are as follows:

(1) This paper proposes a classroom behavior detection model, YOLO-CBD (YOLOv10s Classroom Behavior Detection), targeting crowd occlusion and small-target detection in dense classrooms. By constructing a novel FMBNet backbone, the model enhances the capture of behavior features in densely crowds.
(2) To improve the detection capability for small classroom behaviors in distant instances, the AKConv deformable convolution is incorporated for feature aggregation structure to create the VACSP (VoVGSCSP + AKConv) module, replacing all C2f modules in the neck network. This modification allows the network to more precisely identify and locate occluded small targets, enhancing its handling capacity in complex scenarios.
(3) To address the multi-scale variations of student targets in classroom environments, this study integrates the GSConv module into the Bi-FPN feature pyramid network to resolve the loss of feature diversity after feature fusion. This optimizes the network’s multi-scale features fusion, enhancing its ability to recognize and locate targets of different scales.
(4) This paper modifies CIOU to Wise-IoU, enabling the network to adaptively evaluate the difficulty level of samples by dynamically adjusting the weight function, enhancing the detection performance and generalization ability.

The remainder of this paper is organized as follows: Section 2 describes the related works, and Section 3 provides a detailed introduction on the proposed method. Section 4 describes the experiment setup, and Section 5 provides the various experimental results. Finally, the conclusion is shown in Section 6.

## 2. Related Works

### 2.1. YOLOv10

The YOLOv10 model [28], proposed by researchers from Tsinghua University in 2024, is the latest version of the YOLO (You Only Look Once) series of object detection models. This model inherits the real-time detection advantages of the YOLO series and introduces a consistent dual assignment strategy for training without non-maximum suppression (NMS), achieving efficient end-to-end detection. Compared to previous YOLO models, YOLOv10 is highly scalable and comes in six versions—YOLOv10n, YOLOv10s, YOLOv10m, YOLOv10l, YOLOv10x, and YOLOv10b—each optimized for complex scene detection.

YOLOv10s, with its efficient backbone feature extraction network, neck feature fusion network, and multi-scale detection layer design, demonstrates the best performance in the object detection domain. It is especially suitable for detecting student behaviors in dense classroom environments, as it significantly improves detection accuracy for small targets, providing strong technical support for the development of smart classroom teaching. Therefore, this study uses YOLOv10s as the baseline model, optimizing it for dense crowd behaviors, particularly in cases of occluded behaviors among students in classrooms, significantly enhancing detection accuracy and efficiency. This improvement provides more accurate and reliable technical support for student behavior detection tasks in complex environments. However, YOLOv10 still faces challenges in extracting fine-grained features and maintaining robustness under severe occlusions or irregular spatial distributions, which motivates the architectural improvements proposed in this paper.

### 2.2. Student Classroom Behavior Detection

Student behavior detection is affected by various factors, such as posture, facial expressions, clothing, height, seating, classroom environment, and camera equipment, which can lead to blurred images, cluttered backgrounds, and unclear student movements. This can cause the model to misinterpret student behaviors, making it difficult to distinguish between behaviors. For example, from the machine’s perspective, hand-raising may be confused with face-touching, eye-rubbing, or head-touching, and discussions between students may resemble aimless glancing.

Apparently, if YOLOv10s’s backbone network relies solely on convolutional neural networks (CNNs) for feature extraction, it primarily captures highly localized information, overlooking long-range dependencies within image pixels and limiting the understanding of deep semantic information in complex classroom scenes. CNNs are relatively inefficient at capturing long-distance dependencies. If there is no excessive repetition of convolution operations and multiple layers stacking, long-range semantic interaction cannot be achieved.

EfficientNetV2 [29], a network designed by Google’s team in 2021 for image classification, uses a concurrent structure that blends multi-resolution local and global features, allowing the model to learn more effective weights and enhancing its multi-scale detection performance. Unlike traditional networks, which adjust width, depth, and resolution to improve accuracy, EfficientNetV2 introduces a multi-dimensional scaling method. Firstly, it fixes available network resources at one and uses a grid search to determine the optimal balance of width, depth, and resolution. As resources increase, it proportionally scales these dimensions to ensure optimal balance. Thus, using the EfficientNetV2 architecture as the backbone for image feature extraction can enable a synergistic combination of high-level and low-level features, which help for capturing target features.

### 2.3. Attention Mechanism

Inspired by human perceptual processes, attention mechanisms focus on critical information areas while ignoring non-essential regions, showing excellent performance in various image processing tasks. Typically, attention mechanisms are inserted after convolutional blocks to enhance the ability to handle short- and long-term dependencies and improve learning capabilities for student behavior features in densely occluded scenes.

BiFormer is one of the most popular attention mechanisms, which maintains high internal resolution and merges a SoftMax–Sigmoid combination within the channel and spatial attention blocks [30]. By integrating attention mechanisms into the CNN, BiFormer can automatically learn the importance of different areas, enhancing feature extraction, and ultimately improving classification and detection accuracy.

## 3. Methods

### 3.1. YOLO-CBD Architecture

**Figure 1:**
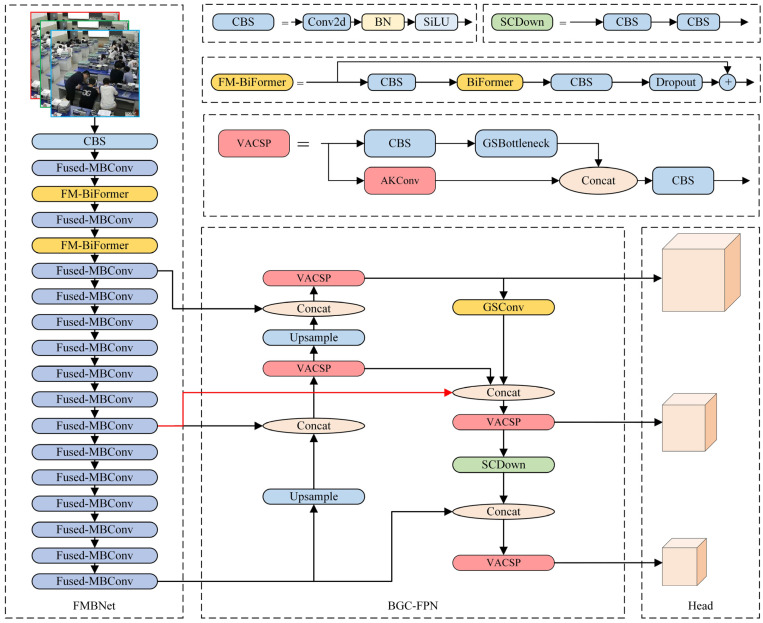
Overall structure of the YOLO-CBD.

As shown in Figure 1, YOLO-CBD consists of the FMBNet backbone network, the BGC-FPN (Bi-FPN + GSConv) neck structure, and the head prediction module, enhancing the model’s capabilities in feature extraction, feature aggregation, multi-scale fusion, and final prediction. To balance detection accuracy and inference speed, the FMBNet backbone is built by integrating the Fused-MBConv module into the baseline YOLOv10s and further enhancing it with the BiFormer attention mechanism at the 3rd and 5th stages. For feature fusion, the BGC-FPN structure is constructed based on VACSP and Bi-FPN [31], improving the integration of semantic and spatial information. In addition, the original CIoU loss used in YOLOv10s is replaced with Wise-IoU, a gradient-friendly loss function that adaptively emphasizes high-quality samples during training. This modification enables smoother gradient descent and better convergence, thereby improving localization accuracy and overall robustness.

The YOLO-CBD model takes input images with a resolution of 640 × 640. First, a 3 × 3 convolution with a stride of 2 reduces the resolution to 320 × 320. This is followed by a Fused-MBConv module that further down-samples the feature map to 160 × 160. For the P3 detection head, a 3 × 3 convolution with a stride of 2 is applied, followed by the FM-BiFormer module to enhance the perceptual capability of the model. The P4 detection head includes another 3 × 3 Fused-MBConv with a stride of 2, reducing the resolution to 40 × 40. Finally, the P5 detection head uses a 3 × 3 Fused-MBConv (stride = 2) to generate a 20 × 20 feature map, enabling accurate small-object detection in densely populated classroom environments.

### 3.2. FMBNet Backbone Network

**Figure 2:**
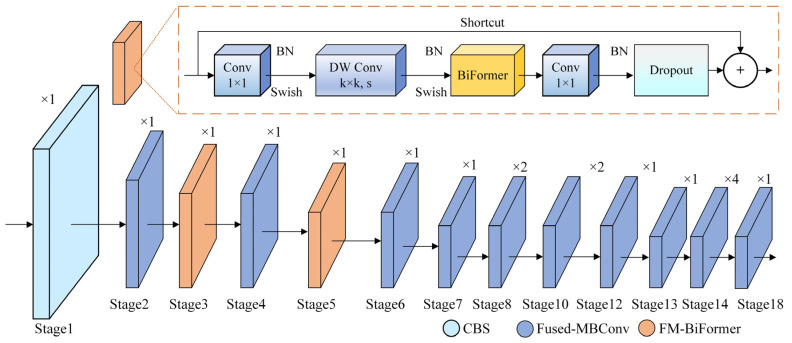
Structure of FMBNet.

EfficientNetV2 improves feature extraction ability in densely occluded scenes by adjusting the convolution modules, effectively avoiding gradient vanishing and high computational costs. Drawing from EfficientNetV2, this paper propose a mixed-scaling method in the FMBNet backbone network to achieve the best scaling factor for width, depth, and input image resolution. The FMBNet structure, comprising a convolution module, multiple Fused-MBConv modules, and FM-BiFormer modules, replaces YOLOv10s’s original backbone network, as shown in Figure 2. The proposed backbone network better balances speed and accuracy, improving the detection performance.

**Figure 3:**
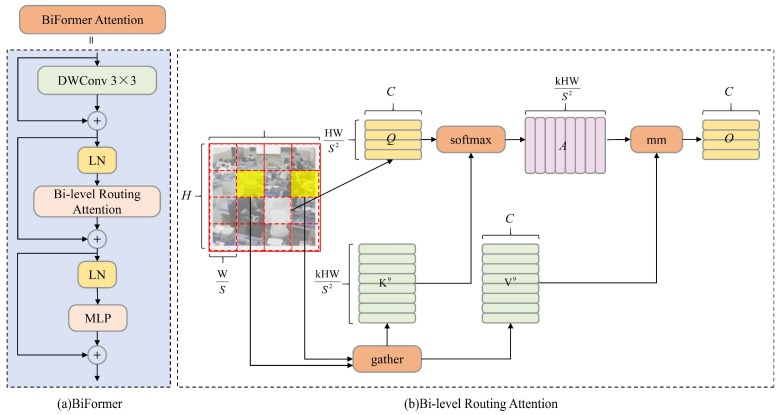
Structure of BiFormer.

In the FMBNet backbone structure, Stage 1 consists of a 3 × 3 convolutional layer with a batch normalization (BN) layer and ReLU6 activation function. Stages 2 to 18 involve stacked Fused-MBConv and FM-BiFormer modules, designed to enhance the network’s feature extraction capability through increased width and depth. Stage 3 integrates a 5 × 5 convolution with the BiFormer attention module to output large-scale features. Stage 5 also uses a 5 × 5 convolution and a BiFormer attention module to output medium-scale features. Stage 18 uses a 3 × 3 convolution layer in the Fused-MBConv module, outputting smaller-scale feature maps. To improve the pedestrian feature recognition in densely occluded environments, the BiFormer attention mechanism is incorporated into the Fused-MBConv modules in Stage 3 and Stage 5. This modification enhances the model focus on key information related to student behavior. Consequently, the extracted features in the FMBNet backbone are significantly enhanced before being fed into the neck network. The BiFormer structure is illustrated in Figure 3.

The operations of the BiFormer mechanism are shown as follows:

Input Image Partitioning: The input image *X*∈*R*^*H*×*W*×*C*^ is divided into *S* × *S* regions, each containing *HW*/*S*^2^ feature vectors. The image *X* is transformed into *X^r^*, which is then linearly mapped to obtain *Q*, *K*, *V*, as shown in Equations (1)–(4).

(1) $$Q=X^{r}W^{q}$$

(2) $$K=X^{r}W^{k}$$

(3) $$V=X^{r}W^{v}$$

(4) $$X^{r},Q,K,V\in R^{S^{2}\times \frac{HW}{S^{2}}\times C}$$

Graph Construction for Region Routing: A directed graph is constructed to route between regions. The average values of *Q* and *K* are computed to yield *Q^r^*, *K^r^*. An adjacency matrix is calculated to represent region-to-region correlations, forming a directed graph to identify participation relationships between different key-value pairs, as shown in Equation (5).

(5) $$A^{r}=Q^{r}{\left(K^{r}\right)}^{T},\;\mathrm{where}\;Q^{r},K^{r}\in R^{S^{2}\times C}$$

Graph Pruning: Each region retains only the top *k* connections by trimming the correlation graph, as described in Equation (6), where the *i*-th row of *I^r^* contains the top *k* most relevant region indices for region *i*.

(6) $$I^{r}=topkIndex(A^{r})$$

Token-to-Token Fine-grained Attention Calculation: Fine-grained attention between tokens is then calculated, as shown in Equation (7).

(7) $$O=Attention(Q,gather(K,I^{r}),gather(V,I^{r}))+LCE(V)$$

Here, *K^g^* = *gather*(*K*, *I^r^*), *V^g^* = *gather*(*V*, *I^r^*) and *LCE*(·) are used with depthwise separable convolution via parameterized values, which is set to 5 for specific performance requirements. By employing this dual-layer routing attention mechanism, BiFormer captures both global and local feature information. Coarse-grained regions filter out unrelated key-value pairs, retaining only relevant regions for fine-grained attention interactions, thus facilitating the retention of a small subset of routing regions. Since this module uses sparse rather than down-sampling techniques, the network focuses more on regions containing small targets and extracts more precise features.

### 3.3. VACSP Module

**Figure 4:**
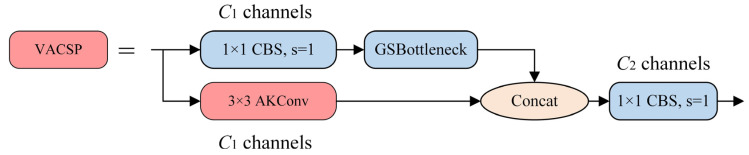
Structure of VACSP.

**Figure 5:**
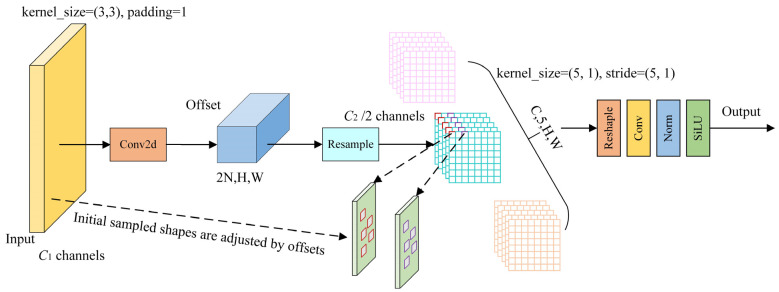
Structure of AKConv.

In the context of detecting student behaviors in complex, densely occluded classroom environments, the YOLOv10s network can be hindered by obstructions, often focusing on local pixel positions rather than capturing essential contextual information. To leverage contextual information, stacking convolutional layers repeatedly would be ideal but poses computational inefficiencies and optimization challenges. This paper proposes a VACSP module, which integrates the VoVGSCSP module [32] and the AKConv module [33] and overcomes these challenges through sparse connections. The structure of VACSP and AKConv are illustrated in Figure 4 and Figure 5.

To enhance spatial adaptability, the AKConv module introduces a dynamic sampling mechanism that adjusts the receptive field according to the content of the input feature map. For a given kernel size, a set of base sampling coordinates P_n_ is first generated using predefined spatial patterns. Then, for each central point P_0_ in the feature map, convolution is applied at the relative positions P_0_ + P_n_ using learned weights. To further improve alignment with objects of varying shapes, AKConv incorporates a learnable offset component that predicts spatial deformations. The final sampling locations are adjusted to P_0_ + P_n_ + Δ, where Δ denotes the predicted offset values. This adaptive mechanism enables the convolution kernel to more accurately focus on semantically relevant regions and improves the extraction of discriminative features, particularly for small or irregularly shaped targets.

The VoVGSCSP module consists of three convolution blocks (CBS) for spatial feature extraction and a GSBottleneck module. As shown in Figure 4, we replace one of the CBS convolution layers with the AKConv module, which enhances the VACSP structure with multi-scale adaptability and higher efficiency by dynamically transforming low-resolution features within each channel. The AKConv module adaptively combines multiple convolution kernels (e.g., 1 × 3, 3 × 1, and 3 × 3) using attention weights derived from global contextual features. This dynamic weighting mechanism allows the network to adjust its receptive field based on spatial content. Compared to the C2f module, AKConv introduces only 0.55 M additional parameters, maintains comparable inference speed in FPS, and improves mAP50 on small-object detection by 2.6%. Specifically, AKConv introduces flexibility into the VACSP structure by initially defining the sampling positions of the convolutional kernels based on target coordinates. It then performs 2D convolutional sampling to extract image features and calculates spatial offsets to adjust these positions. After re-sampling, reshaping, a second convolution, and normalization, the output is activated using the SiLU function. Unlike traditional convolution operations with fixed receptive fields, AKConv modifies the sampling shape for each position, enabling the network to dynamically adapt to image content. This adaptability significantly enhances the network’s ability to focus on small-target regions and extract fine-grained features under complex spatial variations.

### 3.4. BGC-FPN Structure

**Figure 6:**
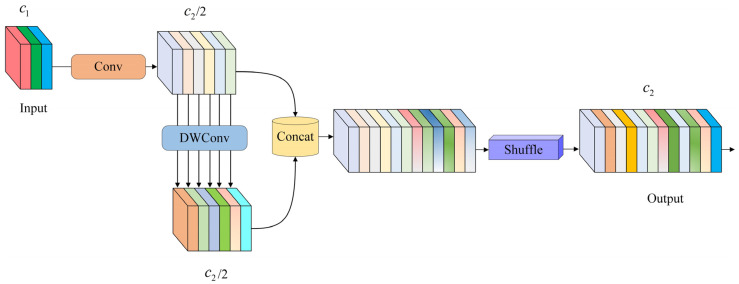
Structure of GSConv.

**Figure 7:**
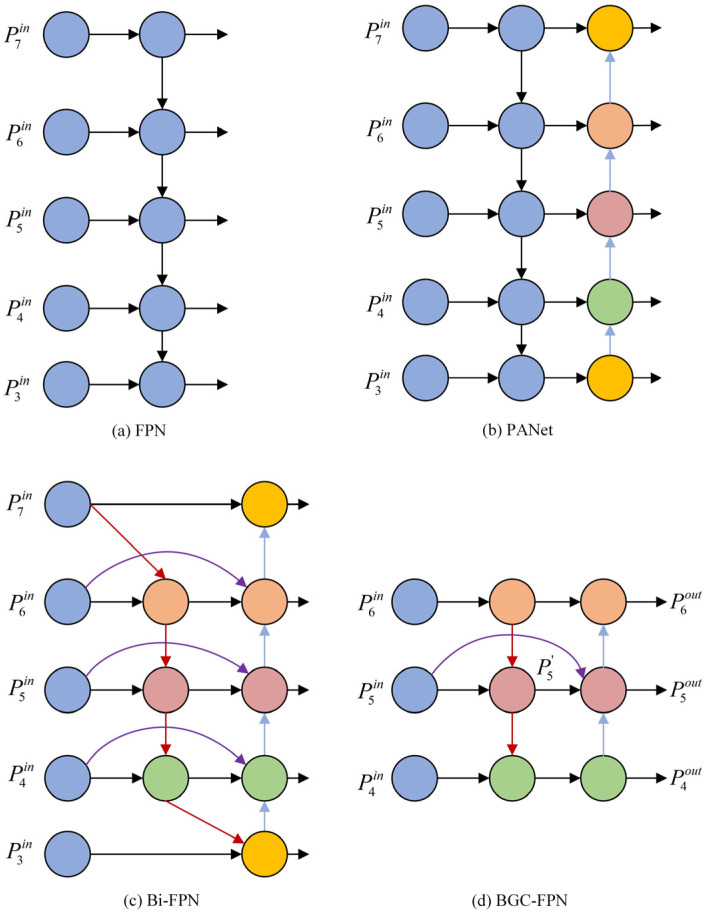
Structure of different feature pyramid networks.

The YOLOv10s model originally adopts the PANet feature fusion structure to enhance fusion performance. However, since all PAN input features are processed by the FPN, PAN lacks some original feature information, which can introduce learning biases and reduce detection accuracy. To improve the detection of attentive behaviors, this paper proposes the BGC-FPN feature pyramid network structure, integrating Bi-FPN’s feature fusion concept into YOLOv10s and replacing the neck’s standard convolution module (CBS) with the GSConv module. GSConv is a lightweight convolution that eliminates the negative impact of channel separation in feature fusion. As illustrated in Figure 6, GSConv first applies standard convolution to the feature map, followed by depthwise separable convolution. The results are concatenated and reshuffled to recombine channels, enhancing model detection performance. The redesigned YOLOv10s neck network, as shown in Figure 7, better suits complex student behavior detection environments.

The BGC-FPN structure differs from Bi-FPN by reducing input nodes to three effective feature layers, suitable for a simpler backbone network. Additionally, an extra edge is added through a skip connection, merging features from the feature extraction network with corresponding features from the bottom-up path, preserving shallow detail information while minimizing deep semantic loss. Compared to PANet, BGC-FPN removes nodes with only one input edge, as they do not undergo feature fusion and contribute minimally to the network. The highlighted purple sections in the structure indicate feature fusions added to increase accuracy within an acceptable range of computational costs. Traditional feature fusion methods, such as concatenation or shortcut operations, simply stack or add feature maps. However, due to varying resolutions among different feature maps, each map contributes differently to fusion. BGC-FPN utilizes a weighted feature fusion mechanism that is fast and efficient for training.

The specific example of BGC-FPN feature fusion is expressed in Equations (7) and (8), illustrating a two-feature fusion at the fifth level.

(8) $$P_{5}^{td}=Conv(\frac{w_{1}\times P_{5}^{in}+w_{2}\times Resize(P_{6}^{in})}{w_{1}+w_{2}+\varepsilon })$$

(9) $$P_{5}^{out}=Conv(\frac{w_{1}^{\prime }\times P_{5}^{in}+w_{2}^{\prime }\times P_{5}^{td}+w_{3}\times Resize(P_{4}^{out})}{w_{1}^{\prime }+w_{2}^{\prime }+w_{3}^{\prime }+\varepsilon })$$

where $P_{5}^{td}$ represents the middle node input the fifth level, $P_{5}^{in}$ is the first node input, *w* is the learned weight parameter, *Resize* is the feature map sampling operation, and *Conv* represents the convolution operation. Overall, BGC-FPN incorporates a weighted bi-directional, cross-scale fusion mechanism, enhancing robustness in multi-scale feature detection for classroom behavior.

### 3.5. Wise-IoU Loss Function

For student classroom behavior detection, the high prevalence of small targets is crucial to the overall detection performance. Traditional loss functions primarily consider the Intersection over Union (IoU) between the predicted and ground truth boxes, but often overlook classification information. By carefully designing a loss function, we can improve model detection accuracy. YOLOv10s utilizes DFL Loss and CIoU Loss for bounding box regression, where CIoU employs a monotonic focus mechanism, but lacks balance between difficult and easy samples. The presence of low-quality samples in the training dataset can negatively impact the detection performance. To address this, we introduce the Wise-IoU loss function [34], which incorporates a dynamic non-monotonic focusing mechanism to balance samples more effectively. Wise-IoU substitutes IoU with an outlier degree assessment of anchor box quality, avoiding excessive penalization due to geometric factors (such as distance and aspect ratio), as shown in Equations (10)–(12).

(10) $$L_{WIoU}=rR_{WIoU}L_{IoU},r=\frac{\beta }{\delta \alpha^{\beta -\alpha }}$$

(11) $$\beta =\frac{L_{IoU}^{*}}{\bar{L_{IoU}}}\in [0,+\infty )$$

(12) $$R_{WIoU}=\exp\left(\frac{{\left(x-x_{gt}\right)}^{2}+{\left(y-y_{gt}\right)}^{2}}{{\left(c_{w}^{2}+c_{h}^{2}\right)}^{*}}\right)$$

Here, *δ* is a threshold parameter that defines the point at which the gradient gain r is maximized (i.e., when *β* = *δ*, *r* = 1). The parameter α controls the curvature and sharpness of the focusing mechanism. *L_IoU_* ∈ [0,1] represents the IoU loss, which will weaken the penalty term for high-quality anchor frames and strengthen their focus on centroid distance in the case of high overlap between anchor and prediction frames, and *R_WIoU_* ∈ [1,exp] represents the Wise-IoU penalty term, which strengthens the loss of ordinary-quality anchor frames. The superscript * stands for not participating in backpropagation, effectively preventing the network model from generating gradients that cannot converge. $\bar{L_{IoU}}$ acts as a normalization factor, representing the sliding average of the increments. *β* stands for the degree of outlier, whose small value implies that the anchor frames are of high quality, and assigns a small gradient gain to them. In addition, smaller gradient weights are assigned to prediction boxes with large outlier values, thereby suppressing the negative impact of low-quality training samples on model optimization. Thus it can focus the bounding box regression loss to the ordinary-quality anchor frames and improve the overall performance of the network.

By balancing penalty terms based on anchor quality, the Wise-IoU function enables the network to adaptively focus on both highly relevant and mid-quality anchor boxes, which is particularly useful in tasks with complex scenes and varied target scales, like student behavior detection in classroom settings.

## 4. Experiment Setup

### 4.1. Dataset and Experimental Environment

The classroom behavior dataset used in this study was collected from various classroom environments. After filtering, a total of 1000 images were retained and split into training, validation, and testing sets using an 8:1:1 ratio through random sampling at the image level. The dataset consists of two common classroom behaviors: focus and distract. These labels were manually annotated by three independent reviewers based on observable behavioral cues.

The real-world classroom environments may exhibit a broader range of behaviors and scenarios. Therefore, to reduce the impact of outliers, we applied extensive data augmentation techniques during training, including random occlusion, brightness jittering, and noise injection, demonstrating the diversity and complexity introduced into the training data.

**Figure 8:**
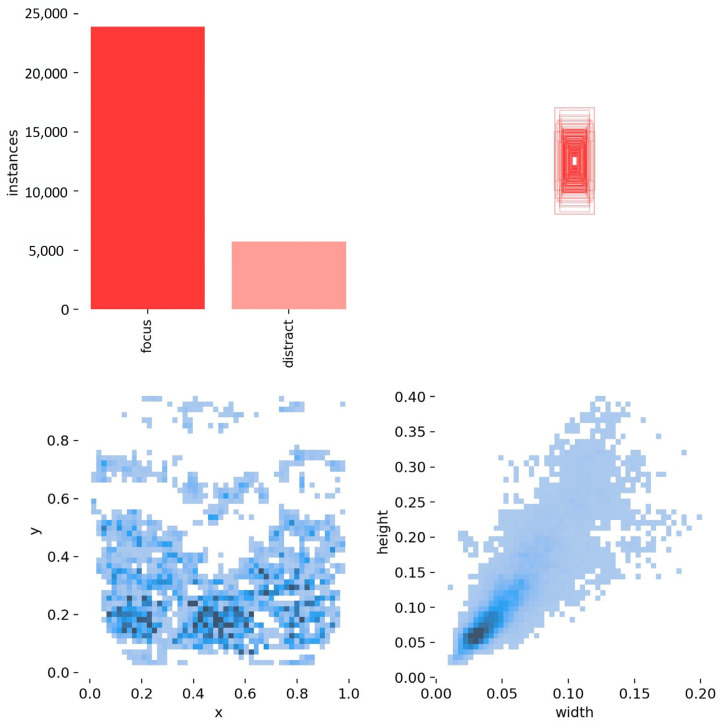
Statistical analysis of the classroom behavior dataset.

To provide a clearer view of the dataset structure, we conducted a statistical analysis of the label distribution, object location, and bounding box dimensions, as shown in Figure 8. The dataset contains approximately 24,000 instances of the ‘focus’ class and 6000 instances of the ‘distract’ class, indicating a class imbalance ratio of approximately 4:1. Heatmaps of object center points reveal that student behaviors are mostly concentrated in the lower-middle region of the image, which aligns with typical classroom camera angles. Furthermore, the width–height distribution confirms that most targets are relatively small, emphasizing the importance of small-object detection in this task. Anchor clustering was also performed to initialize prior boxes that better match the object scales in the dataset.

The experiments were conducted on a system running Ubuntu 18.04 (64-bit) with 64 GB RAM, an NVIDIA GeForce RTX 4090 GPU (24 GB VRAM), and an Intel(R) XeonR Platinum i9-13900k CPU. The model was implemented using the PyTorch 2.0.1 deep learning framework, with CUDA version 11.1 and cuDNN version 8.0.4.

### 4.2. Model Training

**Figure 9:**
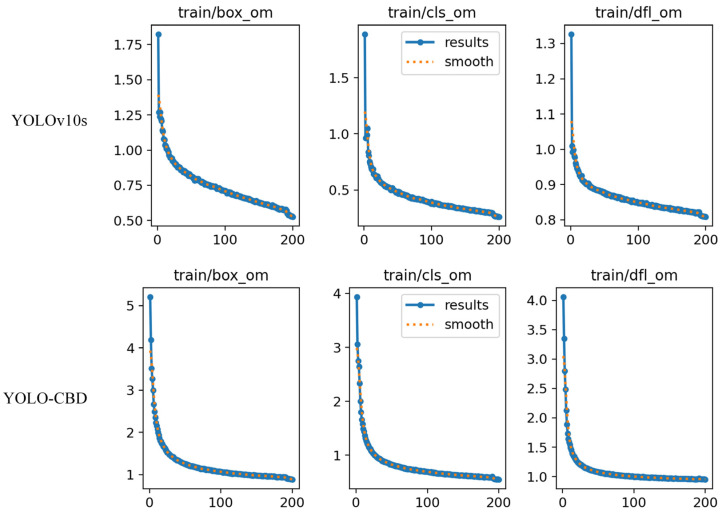
Comparison of loss curves for training YOLOv10s and YOLO-CBD.

The input image size for model training was set to 640 × 640 pixels, with a total of 200 epochs. The batch size and number of workers were both set to 32. The initial learning rate was 0.01, updated using stochastic gradient descent (SGD) with a momentum of 0.937 and weight decay of 0.0005. During training, mosaic data augmentation was used, combining four randomly selected images by flipping and scaling them to create a more diverse visual context. Label smoothing was set to 0.01 to prevent overfitting and improve model generalization. As shown in Figure 9, training results demonstrate that the YOLO-CBD model exhibits a smoother convergence in its loss curve and more stable detection performance compared to the YOLOv10s model.

### 4.3. Evaluation Metrics

The evaluation metrics selected in this study include Precision (*P*), Recall (*R*), mean Average Precision at IoU 0.5 (mAP50), number of parameters (Params), and Frames Per Second (FPS). mAP is the average of the Average Precision (*AP*) values across all categories, providing a more comprehensive evaluation of the model’s performance. Params reflects the model’s size and complexity. The formulas for these metrics are as follows:

(13) $$\mathrm{Precision}=\frac{N_{TP}}{N_{TP}+N_{FP}}$$

(14) $$\mathrm{Recall}=\frac{N_{TP}}{N_{TP}+N_{FN}}$$

(15) $$mAP=\frac{\sum_{i=1}^{m}AP_{i}}{m},\;\mathrm{where}\;AP=\int_{0}^{1}P(r)dr$$

where *TP* (True Positive) represents the number of positive samples correctly identified, *FP* (False Positive) represents the number of positive samples incorrectly identified as negative, *FN* (False Negative) represents the number of negative samples incorrectly identified as positive, and *m* represents the number of object categories being detected. *AP* is the percentage of correctly identified samples out of the total number of samples. By comprehensively evaluating these metrics, this study provides a detailed assessment of the classroom behavior detection method, guiding potential improvements in the detection approach.

## 5. Experimental Results and Analysis

### 5.1. Ablation Study

This study conducted a series of comparative experiments on different attention mechanisms based on the YOLOv10s model. The default SENet attention mechanism in the original Fused-MBConv module was replaced with CA, SimAM, GAMAttention, and BiFormer mechanisms, respectively.

**Figure 10:**
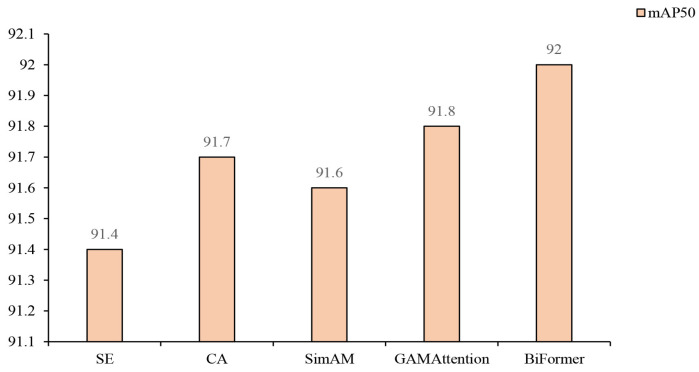
mAP50 values for YOLOv10s using different attention mechanisms.

As illustrated in Figure 10, the YOLOv10s model achieved mAP50 values of 91.4%, 91.7%, 91.6%, and 91.8% when using SE, CA, SimAM, and GAMAttention, respectively. Although all attention mechanisms yielded high detection accuracy, there were slight variations among them. When the BiFormer attention mechanism was employed, the model reached the highest mAP50 value of 92.0%.

To evaluate the effectiveness of Fused-MBConv (A), BiFormer (B), VACSP (C), BGC-FPN (D), and Wise-IoU (E) in classroom behavior detection tasks, this study sequentially introduced new improvement strategies on top of YOLOv10s, constructing five different models. As shown in Table 1, √ indicates the inclusion of a new module, while × denotes the absence of a new module. Each module was added incrementally to the baseline model in a step-by-step manner to systematically assess its individual and cumulative impact on the detection performance.

Table 1 demonstrates the impact on detection accuracy. Incorporating the Fused-MBConv (A), the model improved the P and mAP50 values by 1.6% and 1.5% compared to the original YOLOv10s, indicating enhanced feature extraction for classroom behavior detection. When BiFormer attention was further introduced, P, R, and mAP50 values were, respectively, increased by 3.6%, 1.0%, and 2.1%, proving that the BiFormer attention mechanism (B) can effectively improve the extraction of the upper-body features of distant students and aid in distinguishing student behavior through local features. The introduction of the VACSP module (C) can further improve the P, R, and mAP50 values by 4.5%, 1.9%, and 2.6% over the baseline, suggesting that the VACSP module can enhance feature fusion across different layers and significantly strengthen neck information fusion through global multi-layer feature fusion. With the BGC-FPN (D) addition, The new model achieved gains of 5.3% in P, 3.7% in R, and 3.5% in mAP50, highlighting that BGC-FPN increases the capability to merge multi-scale features, preserving the consistency of classroom behavior features across various scales. After incorporating the Wise-IoU loss function (E), the proposed YOLO-CBD model (Baseline+A+B+C+D+E) saw a further 0.2% increase in mAP50, indicating that Wise-IoU helps fine-tune the bounding box location and size during training, improving detection accuracy. As a result, the proposed model achieved final detection metrics of 93.5% in P, 89.9% in R, and 93.4% in mAP50, showcasing robust detection performance.

Comparatively, the proposed YOLO-CBD model is not the best in terms of Params (9.43 M) and FPS (435). Although the parameter count and inference cost of all improved models increased slightly, YOLO-CBD remains a highly effective detection method in terms of balancing accuracy and efficiency.

**Figure 11:**
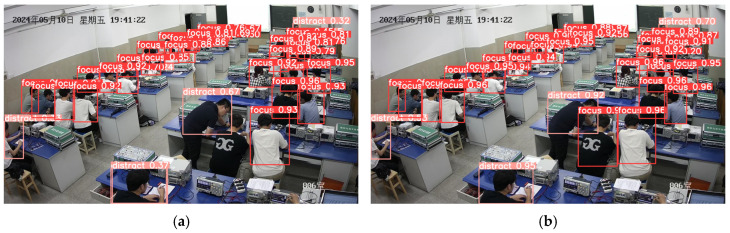
Performance comparison of the baseline and proposed model: (**a**) baseline model; (**b**) proposed model.

Comparison of student classroom behavior detection under dense occlusion and small instances in long-distance images. As shown in Figure 11, we evaluated the detection performance of various models under densely occluded conditions and for distant small instances of classroom behaviors. In Figure 11a, YOLOv10s failed to detect a student who was not paying attention in class, showing a significant issue with missed detections. However, in Figure 11b, YOLO-CBD successfully identified all student behaviors with the highest detection accuracy. In summary, YOLO-CBD demonstrated the detection performance. This indicates that the introduced modules can effectively enhance the ability to extract behavioral features in densely occluded settings and improve multi-scale feature fusion in complex classroom environments.

### 5.2. Visualization Analysis

**Figure 12:**
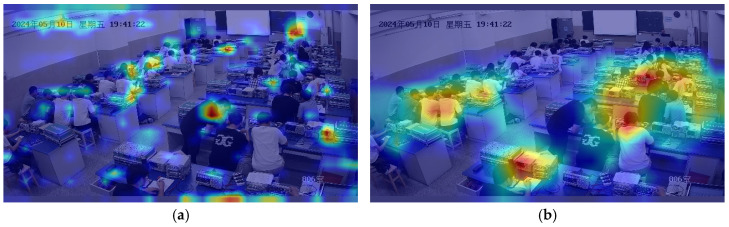
Heat map comparison of the baseline and proposed model: (**a**) baseline model; (**b**) proposed model.

To verify that the proposed YOLO-CBD model focuses on target areas when detecting student classroom behavior under dense occlusion, we applied Grad-CAM [35] technology for visual inspection. Figure 12 presents heatmaps of the baseline model and the proposed YOLO-CBD model under identical conditions. Figure 12a shows the baseline model, where the heatmap reveals a diffuse attention pattern, suggesting insufficient feature-capturing ability and a lack of focus on student targets. Figure 12b shows the YOLO-CBD model, where the detection is precisely centered on student targets, even under occlusion conditions. In summary, the baseline model frequently misidentifies or deviates from the correct detection area under dense occlusion, while the YOLO-CBD model achieves robust location accuracy. This outcome underscores the effectiveness of YOLO-CBD in capturing essential behavioral features, significantly improving detection performance and model interpretability for classroom behavior.

### 5.3. Performance Comparison with Mainstream Models on the Classroom Behavior Dataset

As shown in Table 2, to further verify the detection performance of YOLO-CBD, we compared it with other state-of-the-art models. According to Table 2, YOLO-CBD achieved the highest mAP50 score of 93.4%. Compared to other single-stage object detection networks, such as YOLOv3-spp [13], YOLOv5s [14], YOLOv6s [15], YOLOv7 [16], YOLOv8s [17], YOLOv9-c [18], YOLOv8-Worldv2 [19], YOLOv8-DETR [20], and SSD [21], YOLO-CBD outperformed them by 8.8%, 6.4%, 5.7%, 2.7%, 4.0%, 7.5%, 3.6%, 3.5%, 8.7%, and 28.5%, respectively, in mAP50. Additionally, YOLO-CBD showed clear advantages in Precision (P) and Recall (R) as well.

**Figure 13:**
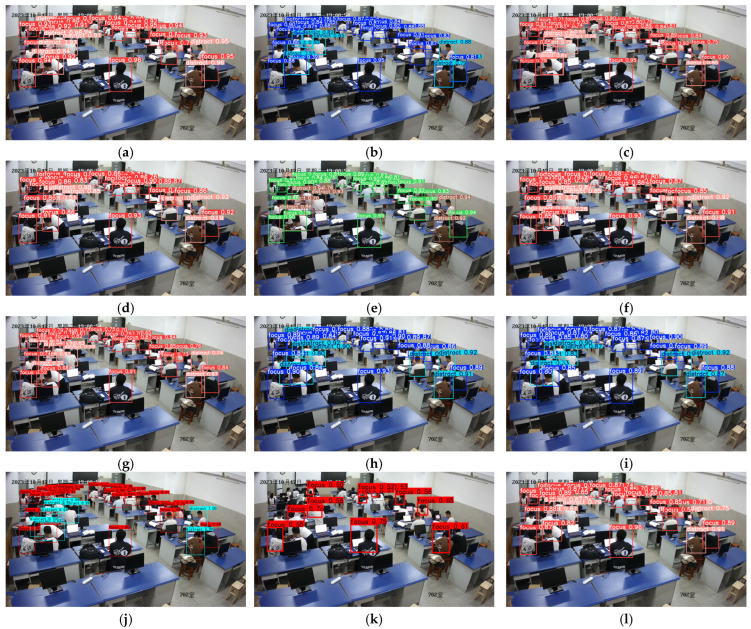
Performance comparison of the mainstream models on the classroom behavior dataset: (**a**) baseline model [28]; (**b**) YOLOv3-spp [13]; (**c**) YOLOv5s [14]; (**d**) YOLOv6s [15]; (**e**) YOLOv7 [16]; (**f**) YOLOv8s [17]; (**g**) YOLOv9-c [18]; (**h**) YOLOv8-Worldv2 [19]; (**i**) YOLOv8-DETR [20]; (**j**) Faster R-CNN [12]; (**k**) SSD [21]; (**l**) YOLO-CBD (ours).

In terms of detection speed, YOLO-CBD reaches 435 FPS, making it faster than YOLOv3-spp, YOLOv5s, YOLOv7, YOLOv9-c, Faster R-CNN, and SSD. Regarding model size, the Params of YOLO-CBD was only 9.43M, which is lower than YOLOv3-spp, YOLOv6s, YOLOv7, and YOLOv8s, YOLOv8-Worldv2, YOLOv8-DETR, and Faster R-CNN, respectively. These characteristics make the proposed YOLO-CBD model more suitable for student classroom behavior detection tasks. In addition, detection results of the mainstream models are shown in Figure 13, which demonstrate the effectiveness of the proposed YOLO-CBD model in terms of vision.

### 5.4. Performance Comparison with Mainstream Models on the Public Dataset

**Figure 14:**
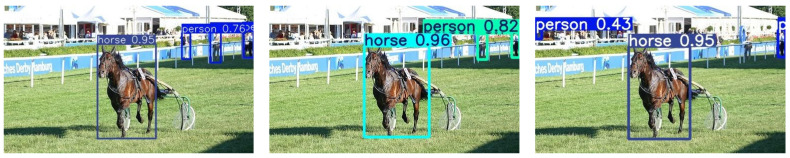

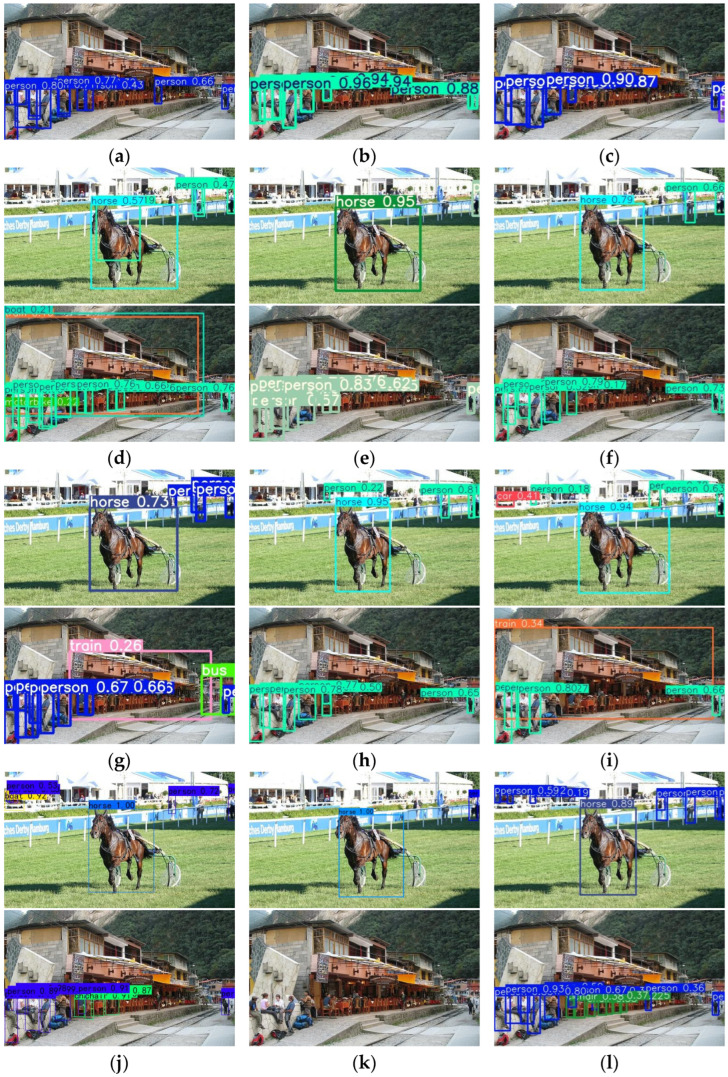
Performance comparison of the mainstream models on the public Pascal VOC2007 dataset: (**a**) baseline model [28]; (**b**) YOLOv3-spp [13]; (**c**) YOLOv5s [14]; (**d**) YOLOv6s [15]; (**e**) YOLOv7 [16]; (**f**) YOLOv8s [17]; (**g**) YOLOv9-c [18]; (**h**) YOLOv8-Worldv2 [19]; (**i**) YOLOv8-DETR [20]; (**j**) Faster R-CNN [12]; (**k**) SSD [21]; (**l**) YOLO-CBD (ours).

To further demonstrate the generalization ability of the proposed YOLO-CBD model, we implemented the experiments on public dataset (Pascal VOC2007 dataset), as shown in Figure 14. It is obvious that YOLO-CBD model is very suitable for detecting small occluded targets at long distances, which demonstrates the effectiveness of the proposed YOLO-CBD model in terms of vision.

In addition, it can been seen from Table 3 that YOLO-CBD achieved the highest mAP50 score of 72.3%. Moreover, YOLO-CBD achieved the optimal balance between detection accuracy, speed, and parameter count.

### 5.5. Experimental Results Analysis and Discussion

Despite the effectiveness of YOLO-CBD in detecting classroom behaviors under occlusion and scale variation, the model still faces several limitations. First, it only supports two behavior classes (focus and distract), which does not fully capture the diversity of student actions in real classroom settings (e.g., writing, hand-raising, discussion). Second, while data augmentation improves robustness, the model may still suffer from degraded accuracy under extreme heavy motion blur or non-frontal camera angles. For example, the persons in the upper right corner of Figure 14l were not detected because the person was too small, which provides insight into potential areas for future improvement.

## 6. Conclusions

This paper proposes a novel classroom behavior detection model, YOLO-CBD, designed to improve detection accuracy and address the challenges of severe occlusion and multi-scale variation in complex classroom environments. Specifically, the BiFormer attention mechanism was integrated into the redesigned FMBNet back-bone to enhance feature extraction under dense occlusion. AKConv was incorporated into the VoVGSCSP module to improve small-target detection at long distances. In the neck network, the BGC-FPN structure was developed by combining Bi-FPN with GSConv, facilitating more effective multi-scale feature fusion and richer semantic information extraction. Additionally, the Wise-IoU loss function was introduced to better handle bounding box localization errors by adaptively focusing on sample difficulty. Experimental results demonstrate that YOLO-CBD achieves an mAP50 of 93.4% with a lightweight model size of approximately 9.43M parameters, meeting real-time detection requirements for classroom behavior analysis.

In future work, we plan to further reduce the model size through model compression techniques such as pruning or knowledge distillation to better meet the deployment requirements on resource-constrained edge devices. Moreover, we intend to expand the range of detected student behaviors to include actions such as writing, discussing, and answering questions, thereby improving the model’s applicability in more diverse smart classroom scenarios.

## Figures and Tables

**Table 1:** Ablation study of YOLOv10s model.

Models	Modules					P (%)	R (%)	mAP50 (%)	Params (M)	FPS
	A	B	C	D	E					
Baseline model	×	×	×	×	×	87.8	86.2	89.9	8.92	435
+A	√	×	×	×	×	89.4	82.2	91.4	9.65	455
+A+B	√	√	×	×	×	91.4	87.2	92.0	9.66	400
+A+B+C	√	√	√	×	×	92.3	88.1	92.5	9.47	435
+A+B+C+D	√	√	√	√	×	93.1	89.7	93.1	9.43	417
YOLO-CBD (ours)	√	√	√	√	√	93.5	89.9	93.4	9.43	435

**Table 2:** Performance comparison of mainstream models on the classroom behavior dataset.

Models	P (%)	R (%)	mAP50 (%)	Params (M)	FPS
YOLOv10s [28]	87.8	86.2	89.9	8.92	435
YOLOv3-spp [13]	83.0	77.6	84.6	62.55	135
YOLOv5s [14]	83.0	81.7	87.0	7.02	333
YOLOv6s [15]	85.0	82.3	87.7	16.30	909
YOLOv7 [16]	85.7	86.2	90.7	36.49	156
YOLOv8s [17]	85.3	85.1	89.4	11.13	909
YOLOv9-c [18]	82.5	79.8	85.9	3.60	132
YOLOv8-Worldv2 [19]	88.1	84.8	89.8	12.75	1250
YOLOv8-DETR [20]	84.4	86.2	89.9	28.79	556
Faster R-CNN [12]	75.3	83.2	84.7	136.71	48
SSD [21]	82.8	40.7	64.9	3.68	146
YOLO-CBD (Ours)	93.5	89.9	93.4	9.43	435

**Table 3:** Performance comparison of mainstream models on the public Pascal VOC2007 dataset.

Models	P (%)	R (%)	mAP50 (%)	Params (M)	FPS
YOLOv10s [28]	74.4	58.8	64.5	8.94	455
YOLOv3-spp [13]	83.9	63.5	71.5	62.65	164
YOLOv5s [14]	76.1	59.6	66.9	7.06	169
YOLOv6s [15]	60.3	47.7	50.6	16.30	476
YOLOv7 [16]	67.3	45.0	49.3	36.58	128
YOLOv8s [17]	63.9	47.7	52.5	11.13	435
YOLOv9-c [18]	52.7	44.2	45.3	3.61	51
YOLOv8-Worldv2 [19]	74.3	60.4	67.1	12.75	333
YOLOv8-DETR [20]	58.8	46.5	48.8	28.80	208
Faster R-CNN [12]	57.6	72.8	70.6	137.08	50
SSD [21]	85.0	54.3	67.1	6.07	135
YOLO-CBD (Ours)	85.2	64.1	72.3	9.44	385

## Data Availability

Data are contained within the article.
